# Forecasting the relative abundance of *Aedes* vector populations to enhance situational awareness for mosquito control operations

**DOI:** 10.1371/journal.pntd.0012671

**Published:** 2024-11-25

**Authors:** Paulo C. Ventura, Allisandra G. Kummer, André B. B. Wilke, Jagadeesh Chitturi, Megan D. Hill, Chalmers Vasquez, Isik Unlu, John-Paul Mutebi, Susanne Kluh, Steve Vetrone, Dan Damian, John Townsend, Maria Litvinova, Marco Ajelli

**Affiliations:** 1 Laboratory for Computational Epidemiology and Public Health, Department of Epidemiology and Biostatistics, Indiana University School of Public Health, Bloomington, Indiana, United States of America; 2 Miami-Dade County Mosquito Control Division, Miami, Florida, United States of America; 3 Greater Los Angeles County Vector Control District, Santa Fe Springs, California, United States of America; 4 Maricopa County Environmental Services Department, Vector Control Division, Phoenix, Arizona, United States of America; University of Queensland & CSIRO Biosecurity Flagship, AUSTRALIA

## Abstract

*Aedes*-borne diseases represent a major public health threat and mosquito control operations represent a key line of defense. Improving the real-time awareness of mosquito control authorities by providing reliable forecasts of the relative abundance of mosquito vectors could greatly enhance control efforts. To this aim, we developed an analytical tool that forecasts *Aedes aegypti* relative abundance 1 to 4 weeks ahead. Forecasts were validated against mosquito surveillance data (2,760 data points) collected over multiple years in four jurisdictions in the US. The symmetric absolute percentage error was in the range 0.43–0.69, and the 90% interquantile range of the forecasts had a coverage of 83–92%. Our forecasts consistently outperformed a reference “naïve” model for all analyzed study sites, forecasting horizon, and for periods with medium/high *Ae*. *aegypti* activity. The developed tool can be instrumental to address the need for evidence-based decision making.

## Author summary

Mosquito control authorities have faced major emergencies, but they lacked access to analytical tools for guiding the planning of mosquito control strategies and outbreak response efforts. This deficiency has not only impeded their situational awareness but has also hindered their ability to make informed decisions regarding intervention planning to respond to *Aedes*-borne disease outbreaks. Our study addresses this need by developing an analytical tool that forecasts *Aedes aegypti* relative abundance 1 to 4 weeks ahead. Our forecasts were consistently accurate and precise for all analyzed study sites, forecasting horizon, and for periods with medium/high *Ae*. *Aegypti* activity. Our results indicate that our analytical tool can be used to guide mosquito control operations, ultimately reducing the risk of *Aedes*-borne disease outbreaks.

## Introduction

Pathogens transmitted by the *Aedes* mosquitos such as dengue virus, Zika virus, and chikungunya virus represent a serious public health threat [1]. The United States are increasingly at risk for *Aedes*-borne diseases having abundant mosquito vector populations [2], thousands of imported cases per year [3], and a history of local outbreaks [4,5]–the largest of which took place in Miami-Dade County, Florida, in 2016 and comprised of 287 (reported) locally transmitted Zika cases [4,6]. Mosquito control operations represent the main means to prevent and control local outbreaks. However, a survey conducted by the National Association of County and City Health Officials has highlighted that the majority of vector control programs in the United States are in need of improvements [7].

One of the identified key areas of improvements is evidence-based guidance to support surveillance and vector control operations [7]. Forecasting methodologies and mathematical modeling have played a central role in improving the decision-making process of health authorities by enhancing situational awareness [8–10]. For instance, the Centers for Disease Control and Prevention (CDC) has been carrying out forecasting initiatives for diseases such as COVID-19 [11] and influenza [12] that have provided forecasts of the number of expected cases, hospitalizations, and/or deaths in a 1 to 4 weeks’ time horizon. These have been used to make informed evidence-based decisions about control strategies (such as mask mandates and school closures) and preparedness planning for the healthcare system. However, instruments for short-term forecast of the relative abundance of mosquito vector populations are still lacking, and the decision-making process of mosquito control authorities would highly benefit from them. In fact, these tools are instrumental to improve the situational awareness of mosquito control authorities and other stakeholders, which would allow for better planning of mosquito surveillance and control operations.

The aim of this study is to develop an analytical tool that provides real-time forecasts of *Ae*. *aegypti* relative abundance. The proposed methodology is validated against mosquito surveillance data for multiple years collected in four jurisdictions in the southern US (Los Angeles County, CA, Maricopa County, AZ, Key West, FL, and Miami-Dade County, FL). These jurisdictions have highly different climatic conditions ranging from subtropical (Key West and Miami-Dade County) to arid (Maricopa County) to temperate (Los Angeles County) and three of them have a history of *Aedes*-borne disease transmission: Miami-Dade County (dengue and Zika), Key West (dengue), and Maricopa County (dengue) [5].

## Results

### Analytical forecasting tool

**Fig 1:**
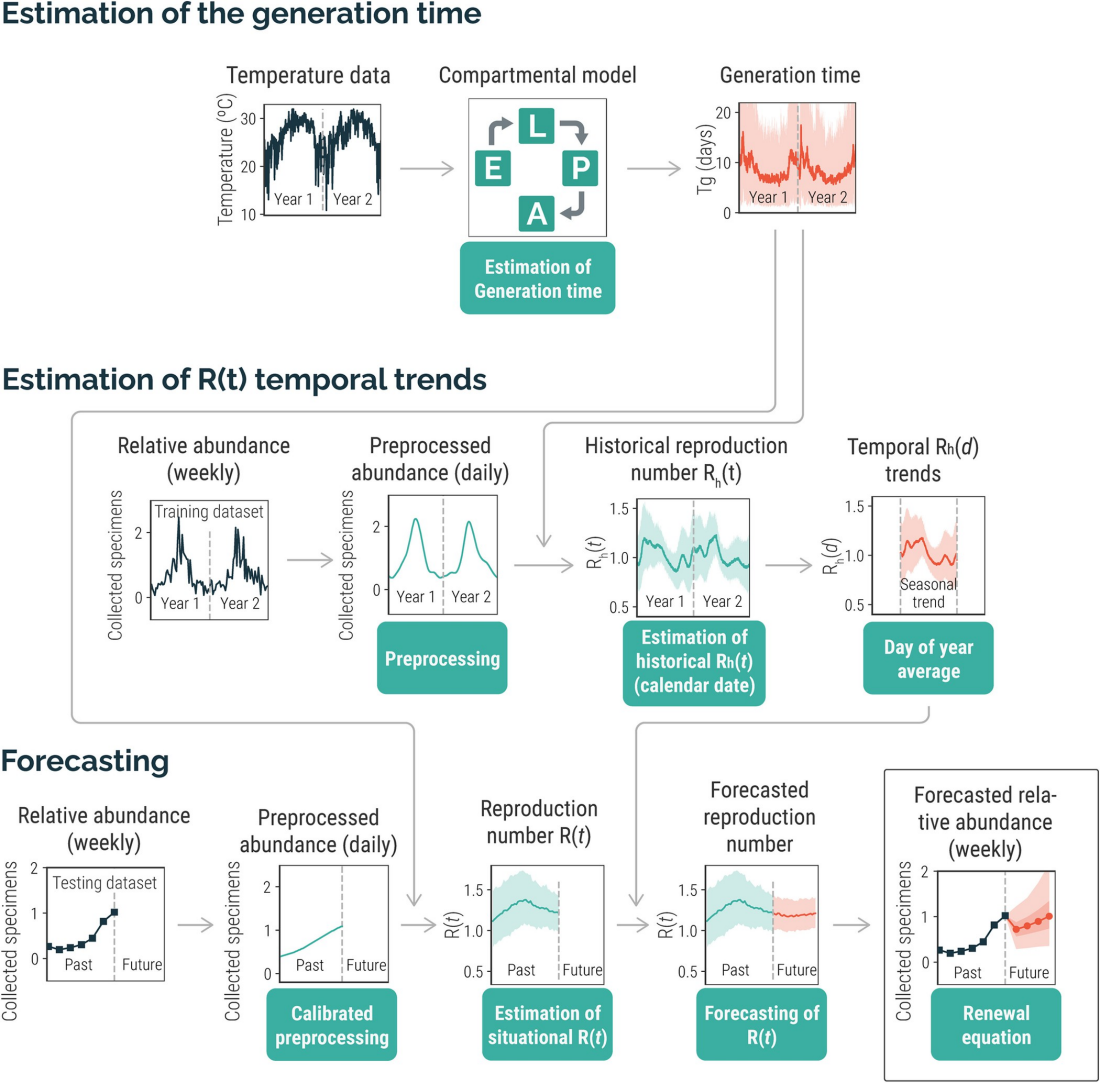
Schematic representation of the developed analytical forecasting tool.

We developed a three-step procedure to forecast the relative abundance (i.e., number of mosquito specimens collected by the mosquito surveillance system) of *Ae*. *aegypti* for a time horizon of 1 to 4 weeks (see Fig 1 for a conceptual representation of the developed analytical forecasting tool). For each study location, the procedure works as follows:

1. For each analyzed year, we relied on historical temperature data, and temperature-dependent mortality and development rates for the different stages of the *Ae*. *aegypti* lifecycle to estimate the generation time (i.e., the average time between two consecutive generations of mosquitoes). This step is carried out through the simulation of a mechanistic compartmental model of the *Ae*. *aegypti* lifecycle.
2. For each analyzed year of the training dataset, we relied on historical mosquito surveillance data aggregated at the jurisdiction level and our estimates of the generation time (from step 1) to estimate historical temporal trends of the net reproduction number (i.e., the average number of female mosquitoes generated by a female at time *t*). We refer to this quantity as the *historical reproduction number*, which we estimated through a Bayesian approach.
3. For the year of the forecast, we use the same Bayesian approach of step 2 to estimate the reproduction number until the forecast date (i.e., “today”, if the approach were to be applied in real time); we combine this estimate with the historical reproduction number (from step 2) to obtain quantile-based forecasts of the reproduction number. We then input these forecasts to a renewal equation to forecast the relative abundance of *Ae*. *aegypti* at the jurisdiction level.

Details on the developed methodology are reported in the Methods section.

To validate 2,760 individual forecast datapoints against empirical mosquito surveillance data aggregated at the jurisdiction level, we used several commonly adopted metrics (see Methods). In the main text, we report the results for multiple metrics of forecast accuracy and coverage. To measure the accuracy, we relied on the symmetric absolute percentage error (sMAPE [13]) and the Weighted Interval Score (WIS [14]). The coverage counts the percentage of datapoints that lie within the interquantile range (IQR) of the forecast; for example, a coverage of 90% is the target result for the 90% IQR forecast [15]. To provide a context to the performance of our forecasting tool, we adapted a frequently used “naïve” model to forecast the *Ae*. *aegypti* abundance [11] (see Methods for details). The same evaluation metrics are used to evaluate the performance of both models.

### Forecasting *Ae*. *aegypti* relative abundance

**Fig 2:**
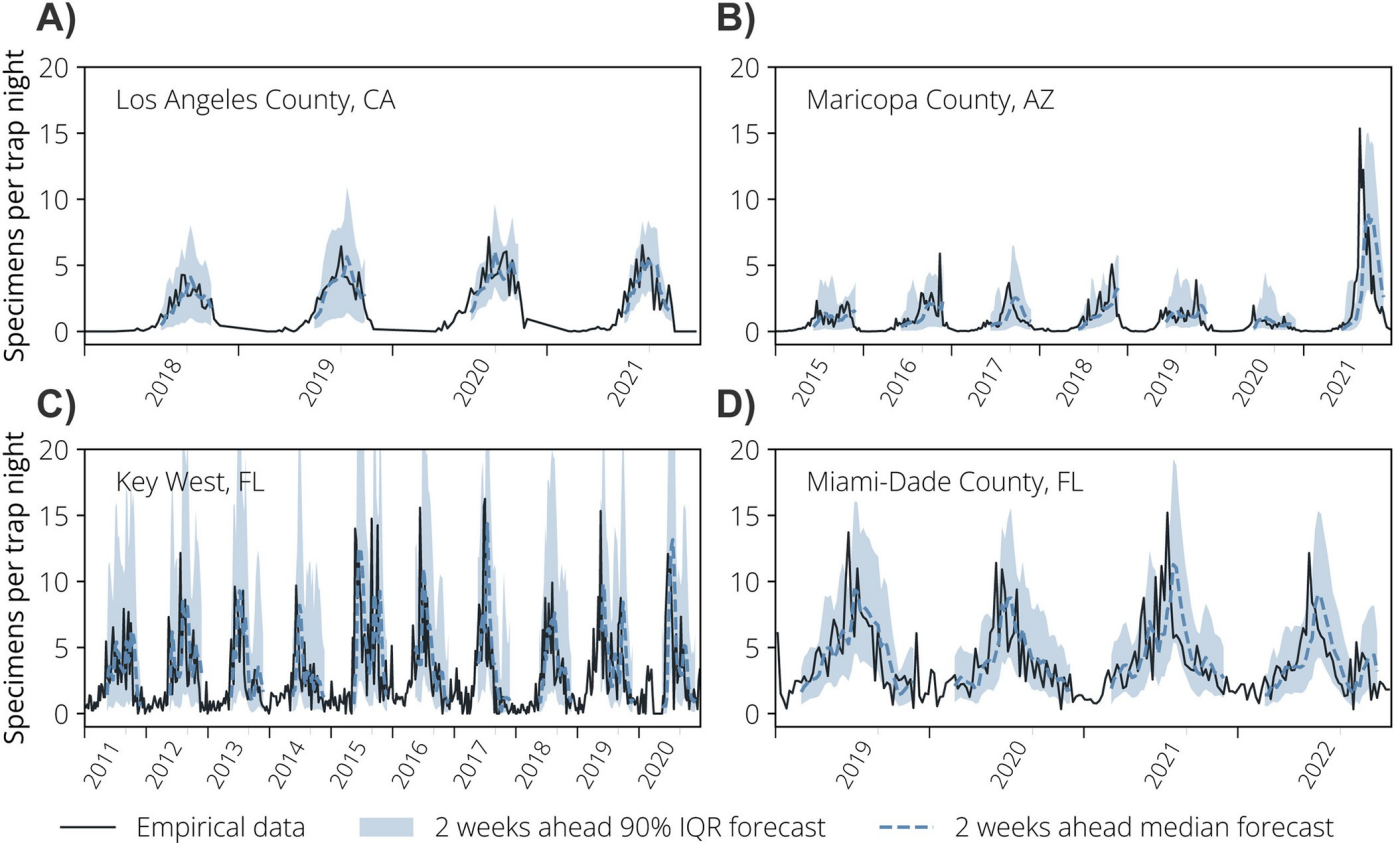
Mosquito relative abundance over time. **A** Observed and forecasted number of collected specimens per trap night over time in Los Angeles County, CA. The solid line represents the number of *Ae*. *aegypti* collected by the mosquito surveillance system per week, the dashed line represents the median of the second week of the 2-week ahead forecast, and the shaded areas are the 90% IQR of the second week of the 2-week ahead forecast. **B** As A, but for Maricopa County, AZ. **C** As A, but for Key West, FL. **D** As A, but for Miami-Dade County, FL, respectively.

The mosquito surveillance data showed pronounced seasonal patterns along with substantial short-term fluctuations. The median 2-week ahead forecast results for all study sites were able to capture both seasonal trends and the complex patterns of the empirical data, with the 2-week ahead forecast 90% interquantile range (90% IQR) covering the majority of the data points (Fig 2; the results for the 1-week, 3-week, and 4-week ahead forecasts are shown in Figs B, C and D in S1 Text).

In Los Angeles County, CA, the *Ae*. *aegypti* active season was notably shorter compared to the other study sites. The longest period of activity occurred from July to December of 2020, spanning 7 months. The highest activity was recorded in 2020, with a peak of 7.14 captured specimens per trap night in a single week. The median 2-week ahead forecast effectively captured the trends in the data, encompassing both the seasons with the lowest (2018) and the highest (2020) activity, and yielding an overall sMAPE (symmetric absolute percentage error, see Methods for details) score of 0.30, which was the smallest error among all study sites. Furthermore, the 90% IQR of the 2-week ahead forecast accurately captured seasonal trends and short-term oscillations, precisely encompassing 90.0% of the observed values (Fig 2A).

Maricopa County, AZ, exhibited a diverse seasonal pattern with multiple peaks and prolonged *Ae*. *aegypti* activity. Notably, activity generally peaked towards the end of the season (November), except for 2020 (the first year of the COVID-19 pandemic) when activity peaked at the beginning of the season, resulting in overall low activity levels. Our forecasting approach effectively adapted to this diversity in seasonal patterns (Fig 2B) with an overall sMAPE of 0.54 for the median of the 2-week ahead forecast. The 90% IQR of the 2-week ahead forecast had an overall coverage of 82.7%, capturing a majority of data points. Nonetheless, our approach missed some steeper fluctuations, particularly notable during the 2021 season, which exhibited unusually high activity (Fig 2B).

Key West, FL, showed the largest activity peaks, reaching the maximum of 16.3 captured specimens per trap night in 2017. The variability between seasons was comparatively smaller than in other locations, although secondary surges were observed in 2015 and 2019. The median of the 2-week ahead forecast had a sMAPE of 0.62, representing the largest error among the four locations. On the other hand, the 90% IQR of the 2-week ahead forecast consistently captured the oscillations in the data, reaching a coverage of 91.9%, the highest among locations.

Miami-Dade County, FL, had the longest *Ae*. *aegypti* activity seasons among all locations, spanning approximately 9 months of intense activity annually. We observed a single peak for most seasons, typically occurring around the month of July, with an exception in 2022 where we observed a secondary surge around October. The median of the 2-week ahead forecast accurately followed the trend of the data, yielding an overall sMAPE of 0.38. The 90% IQR of the 2-week ahead forecast captured 87.9% of the data points (Fig 2D).

**Fig 3:**
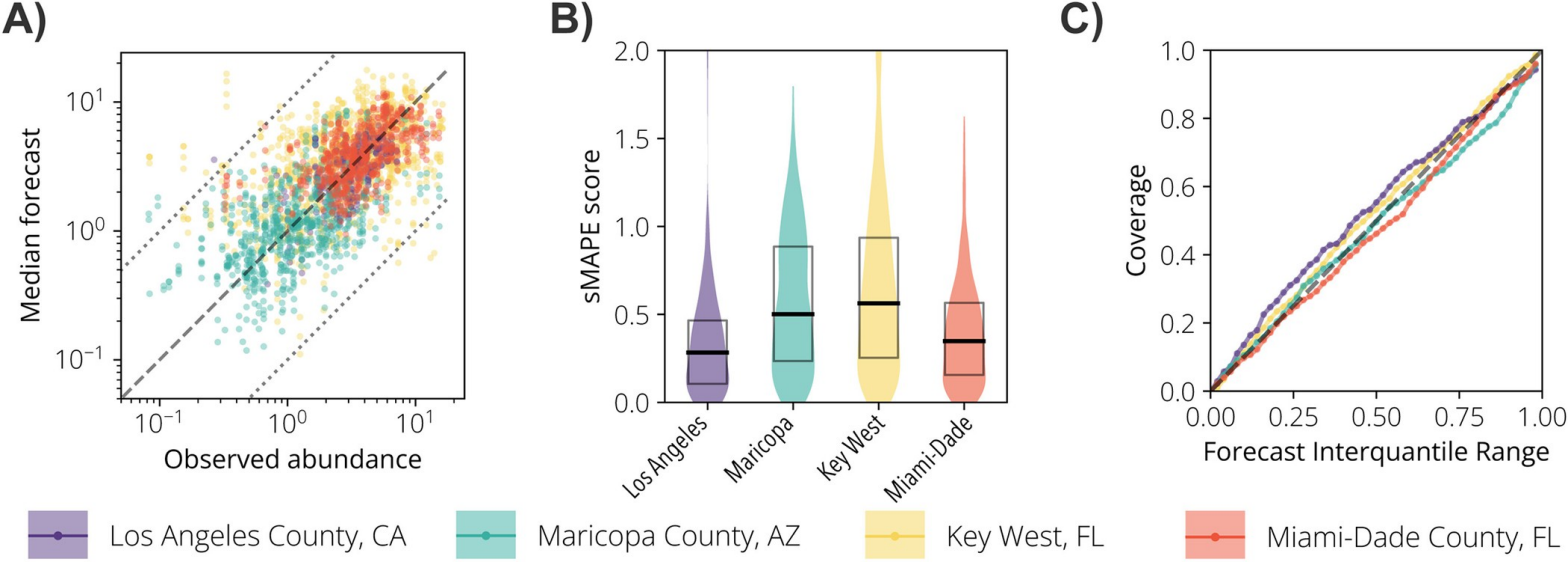
Forecasts accuracy and coverage. **A** Scatter plot of the observed vs. median forecasted *Ae*. *aegypti* relative abundance for each study site and week. All forecasting horizons (1- to 4-week ahead forecasts) are included in this plot. Each color represents a study site. The dashed line is the identity line, and the dotted lines represent the variability within one order of magnitude. **B** Violin plot representing the distribution of the sMAPE for each study site. The boxes report the 25%, 50%, and 75% quantiles of sMAPE. **C** Forecast coverage as a function of the forecast interquantile range. Each colored line represents a study site. The dashed line is the identity line.

Overall, considering 1- to 4-week ahead forecasts, the median forecasts were symmetrically distributed around the observed values– 54% of overestimations against 46% underestimations–with 97.7% of the forecasts within the same order of magnitude as the empirical observations (Fig 3A). Key West showed the highest sMAPE between the study sites (Fig 3B). Regarding the forecast’s coverage, the performance difference among the study sites was marginal (Fig 3C). For instance, the 90% IQR of the forecasts had a coverage between 83% (Maricopa County, AZ) and 92% (Key West, FL), and the 50% IQR had a coverage between 46% (Miami-Dade County, FL) and 55% (Los Angeles County, CA). For all study sites, our forecasting tool outperformed the naïve model in terms of WIS (see methods for details on this metric) and coverage (Table A in S1 Text). For Maricopa County, the naïve model shows a better sMAPE than our tool, primarily due to the subpar performance of our forecasting tool during the atypical 2021 season.

### Forecast accuracy and coverage by time horizon

**Fig 4:**
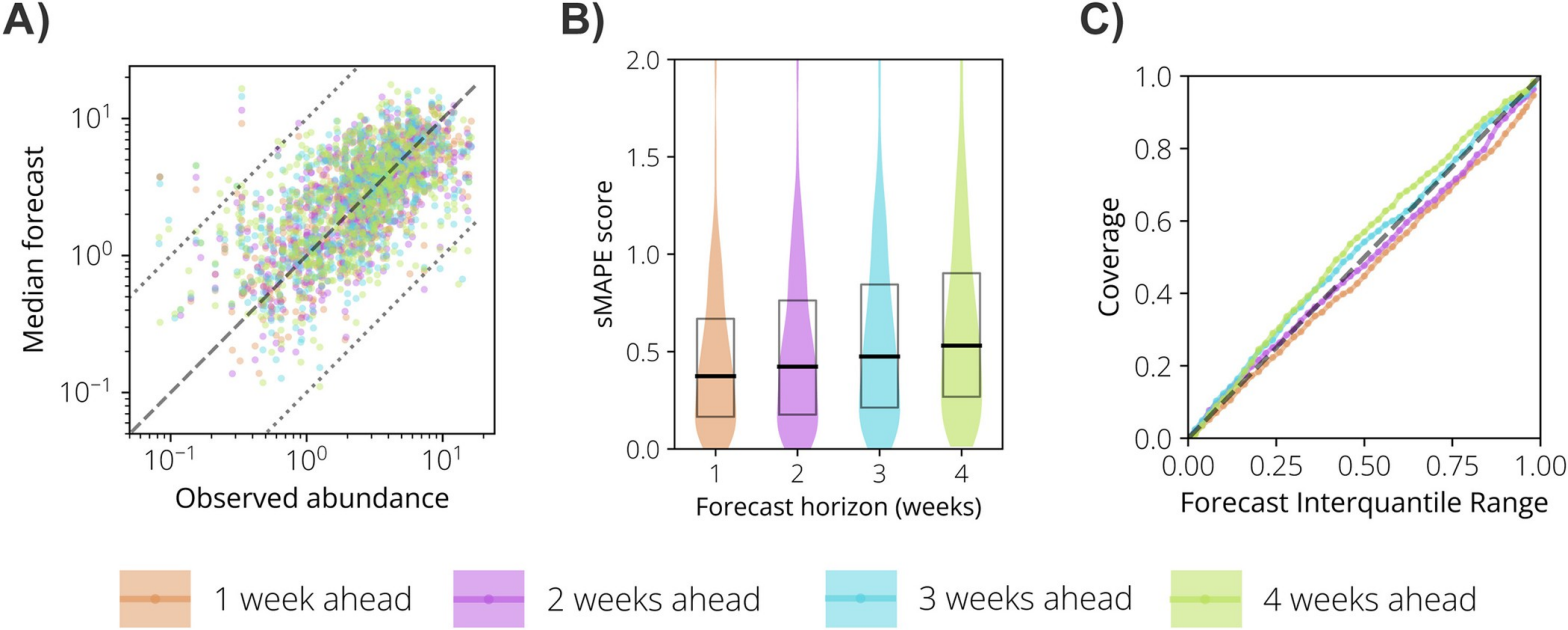
Mosquito abundance forecast results for each forecast horizon week. **A** Scatter plot of the observed vs. median forecasted *Ae*. *aegypti* relative abundance for each study site and week. Each color represents a forecasting horizon in weeks ahead of the reference date. The dashed line is the identity line, and the dotted lines represent the variability within one order of magnitude. **B** Violin plot representing the distribution of the sMAPE for each forecasting horizon in weeks. The boxes report the 25%, 50%, and 75% quantiles of sMAPE. **C** Forecast coverage as a function of the forecast interquantile range. Each colored line represents a forecasting horizon. The dashed line is the identity line.

Our forecasting tool performs well over the four weeks of horizon (Fig 4A), although we observe a slight decrease in the accuracy of median forecasts for longer forecasting windows. Specifically, the sMAPE for all locations and seasons increased from 0.37 for the 1-week ahead forecast to 0.53 for the 4-week ahead forecasts (Fig 4B). Despite that, the coverage of our forecasts displays a slight increase from earlier to later forecast horizons, going from 84% to 93% for the 90% IQR forecasts and from 45% to 57% for the 50% IQR forecasts (Fig 4C). Our forecasting tool also had better performance than the naïve model through the four weeks of the forecast horizon (Table 1). Measured by the WIS, our tool outperformed the naïve model for the 1, 2 and 3 weeks ahead forecast, with the best relative score at the 1-week horizon and the lowest relative score for the 4-week horizon. For the sMAPE and the 95% coverage error, our tool had better performance in all forecast horizons.

**Table 1:** Performance of our forecasting tool with respect to a naïve model. The first data column shows the season average WIS score of our model divided by the same score of the naïve model. The second data column shows the season sMAPE score of our model divided by the same score of the naïve model. For both these columns, a ratio smaller than 1 means that our model had better performance than the naïve model. The third data column shows the absolute 95% coverage error of our forecasting tool subtracted by the same score of the naïve model. For this column, a negative value means that our model performed better than the naïve in terms of coverage. Cells with a green background represent those where our forecasting tool performs better the naïve model; for red cells the opposite is true.

		Season normalized WIS (ratio)	sMAPE score (ratio)	95% coverage error (diff)
**Study site**	**Key West, FL**	0.999	0.938	-10.6%
	**Los Angeles County, CA**	0.684	0.782	-32.9%
	**Maricopa County, AZ**	0.961	1.136	-16.9%
	**Miami-Dade County, FL**	0.879	0.926	-3.0%
**Forecast horizon**	**1 week ahead**	0.887	0.995	-12.6%
	**2 weeks ahead**	0.899	0.960	-12.9%
	**3 weeks ahead**	0.943	0.962	-13.9%
	**4 weeks ahead**	1.029	0.967	-12.0%
**Mosquito activity**	**Low activity**	1.163	1.054	1.1%
	**Medium activity**	0.953	0.917	-11.2%
	**High activity**	0.891	0.970	-26.5%

### Forecast accuracy and coverage by *Ae*. *aegypti* activity level

**Fig 5:**
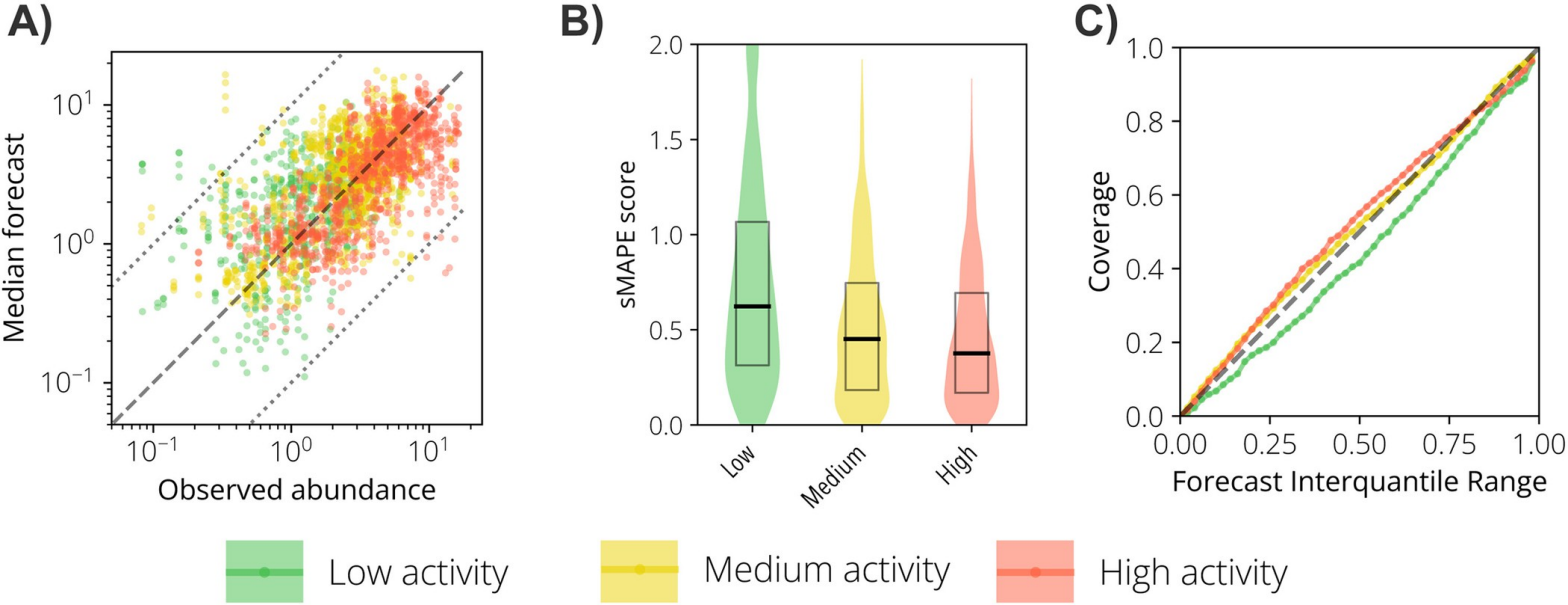
Mosquito abundance forecast results by mosquito activity levels. **A** Scatter plot of the observed vs. median forecasted *Ae*. *aegypti* relative abundance for each study site and week. Each color represents a mosquito activity level (low, medium and high). The dashed line is the identity line, and the dotted lines represent the variability within one order of magnitude. **B** Violin plot representing the distribution of the sMAPE for each activity level. The boxes report the 25%, 50%, and 75% quantiles of sMAPE. **C** Forecast coverage as a function of the forecast interquantile range. Each colored line represents an activity level. The dashed line is the identity line.

We stratified the observations by three activity levels: low (i.e., observations are lower than 30% of the maximum value observed in that season), medium (i.e., observations between 30% and 70% of the maximum value observed in that season), and high (i.e., observations above 70% of the maximum value observed in that season). By using this stratification, we found that the median forecasts were more accurate for medium and high activity periods (Fig 5A). Specifically, sMAPE increased from 0.46 for high-activity periods to 0.52 for medium-activity periods and 0.74 for low-activity periods (Fig 5B). In addition, the coverage for medium and high mosquito activity was consistently higher than for low activity for all interquantile ranges (Fig 5C). As compared to the naïve model, our tool showed much superior performance for medium and high activity periods according to all metrics; however, the naïve model outperformed our tool during low activity periods (Table 1).

## Discussion

Pathogens transmitted by *Aedes aegypti* pose a serious public health threat. Controlling the mosquito vector population is the first line of defense to limit their transmission, but it does entail major challenges. Forecasting tools can provide public health authorities with better situational awareness to make informed decisions on mosquito control strategies. However, most of the current forecasting efforts are focused on vector presence [16] or on the incidence of already endemic mosquito-borne diseases such as the West Nile fever in the USA [17]. Here we have developed a forecasting tool that provides quantitative estimates of *Ae*. *aegypti* relative abundance over a 1- to 4-week ahead period. Our approach relies on estimating the value of the population reproduction number. The reproduction number encompasses socio-environmental determinants into a single indicator which changes over time due to temporal variations in environmental factors such as temperature, precipitation, implemented vector control interventions, hosts availability, which translates into a different reproduction potential and mortality rate of the mosquito population. The developed tool is very parsimonious regarding data input as it is based only on data on past relative abundance of *Ae*. *aegypti* and historical temperature data. The method has been validated on 25 distinct seasons comprising 2760 individual forecasts, distributed across four jurisdictions in the US with highly different climatic conditions. We have shown that our method consistently provides reliable forecasts over four weeks, with reliable performance during the active seasons for *Ae*. *aegypti*.

The data collected from each study site had unique features due to climatic, environmental, and socio-economic differences as well as mosquito collection methods. For example, Miami-Dade County and Key West, FL, displayed modest variability between seasons, while Maricopa had seasons with highly different trends. Moreover, the relative abundance of *Ae*. *aegypti* is remarkably different between study sites, with Key West and Miami-Dade County consistently showing peak activity with over 10 collected specimens per trap night, while Maricopa County and Los Angeles County rarely exceed 5 collected specimens per trap night. Nevertheless, our forecasting tool adapted to these particularities as shown by our extensive validations using multiple performance metrics. Our comparison between study sites shows that the sMAPE score of the two-week horizon median forecast had an overall minimum of 0.30 (most accurate) and a maximum of 0.62 (least accurate) for Los Angeles County, CA, and Key West, FL, respectively. The overall coverage of the 90% IQR forecast was the largest for Key West, FL, at 91.9% and the lowest for Maricopa County, AZ, with 82.7% of points captured. The coverage of all IQR intervals was very consistent between locations, meaning that the methodology was effective for all study sites. Although the performance of our method was overall consistent between seasons and locations, there were exceptions. For example, the 2021 season in Maricopa showed an atypically high and anticipated peak; our forecast showed a delay in catching the timing of this peak. Overall, our forecasting tool performs well in most circumstances we tested, and it consistently outperforms a reference naïve model, with the exception of low mosquito activity periods. Our results show that the performance of our forecasting tool improves proportionally to the density of the *Ae*. *aegypti* population, suggesting that the performance would further improve in areas with higher mosquito density.

To limit the risk of local outbreaks of *Aedes*-borne diseases, it is important to: i) early detect possible surges in mosquito vector populations [18], ii) focus control efforts during periods when the effectiveness of interventions is optimal [19], and iii) act timely, with mosquito control activities carried out on a regular basis [20]. In these regards, our forecasts were reliable for up to four weeks ahead. In fact, although the accuracy of the median forecast slightly decreased over the time (consistent with previous findings for infectious disease forecasts [11,12]), the coverages were substantially similar between different time horizons, suggesting that our approach is able to capture uncertainties and fluctuations uniformly over the time of the forecast. Overall, these findings support the possible use our forecasting approach as a tool to improve mosquito control decision making.

Our forecasting tool is based on the dynamic estimation of the reproduction number of the mosquito population, which is affected by changes in the environment (e.g., temperature, precipitation) as well as the effect of interventions. Therefore, in principle, the effect of vector control measures is taken into consideration in our forecasting tool. Nonetheless, it is important to stress that vector control measures in the four study sites are carried out in well-defined areas (e.g., neighborhood, block) in specific days and are not used indiscriminately. As such, we expect them to have a moderate impact on mosquito collections aggregated at the jurisdiction level. Nonetheless, it would be important to systematically analyze the performance of our forecasting tool in the absence vs. presence of interventions. This would be especially relevant for local-level (e.g., neighborhood) forecasts.

It is important to stress that previous efforts were successfully made into estimating mosquito presence and (relative) abundance based on environmental (e.g., temperature, rainfall, vegetation) and socio-economic (e.g., income, population density) variables [21–28] as well as mosquito surveillance data [22,24,28]. Such approaches provide critical information for estimating the determinants of the transmission risk and dynamics of mosquito-borne diseases. Our work, on the other hand, provides estimates of the relative mosquito abundance in the upcoming 4 weeks based on today’s evidence. In other words, our approach does not aim to provide a general understating of mosquito population dynamics or long-term patterns, but it is intended to provide mosquito control authorities with actionable knowledge on the short-term relative abundance of the analyzed mosquito population. Similar efforts have been made in the broader context of mosquito-borne diseases. For example, the CDC has led several initiatives focusing on peak incidence, the week of the peak, and total incidence of dengue [29], the total number of West Nile Virus (WNV) neuroinvasive disease cases in a calendar year at the county level [30], and monthly number of WNV neuroinvasive disease cases at the state level [31], and a DARPA initiative has focused on chikungunya incidence [32]. In the specific context of forecasting mosquito vector populations, the CDC has led an initiative aimed at forecasting the presence of *Ae*. *aegypti* or *Ae*. *albopictus* in US counties by month [33]. However, differently from our study, that initiative focused only on the presence of mosquito vector populations rather than on providing short-term forecasts of the relative abundance.

Our study is subject to several limitations. First, although the overall performance of our forecasts was satisfactory, there is room for improvement for seasons with atypical patterns such as the 2021 season in Maricopa County. Similar to what found for infectious diseases forecasting efforts [11,12], our approach is relatively slow to adapt to atypical and abrupt changes in the dynamics. Second, our forecasting approach is based on the analysis of the mosquito surveillance data from the forecasted year and at least one past year–we relied on 2 years for Los Angeles County, 3 for Maricopa County, 5 for Key West, and 2 for Miami-Dade County. Although this is not a very demanding requirement, our approach would need to be adapted for jurisdictions with newly established mosquito surveillance systems. Moreover, we did not investigate whether there exist an optimal number of past seasons to be analyzed to calibrate our approach. Third, the data used for our analysis is aggregated at the jurisdiction level, which does not allow for identifying geographical heterogeneities in mosquito abundance. Future analyses should consider incorporating this variability into the modeling framework. Fourth, although our forecasting tool can in principle be used for other mosquito species, we tested it only on *Ae*. *aegypti*. A possible challenge to extend our approach to other species is that it relies on the availability of temperature-dependent mosquito development and mortality rates, which may not be available for every species. Fifth, we currently assume that the generation time during the forecasted period is constant and equals to the average over the last 7 days. The integration of weather forecast data on temperature may allow dropping this assumption, possibly enhancing the accuracy and/or coverage of our forecasts. Sixth, our analysis was conducted at the jurisdiction level (i.e., county or city). Forecasts at a smaller spatial resolution (e.g., neighborhood or sub-neighborhood level) may provide valuable information to mosquito control authorities to plan control efforts. In this regard, we would like to emphasize that our forecasting approach was able to provide solid forecasts for Key West, whose surveillance system comprises only 12 traps and the number of collected *Ae*. *aegypti* specimens per week rarely exceeds 100 over the course of a season. This suggests that it may be possible to sub-divide counties with larger number of traps and collected specimens (as it is the case in Los Angeles County, Maricopa County, and Miami-Dade County) into smaller areas and use our forecasting approach on each of them separately.

In conclusion, we have developed and validated a tool for the real-time forecasting of mosquito relative abundance. Our forecasting tool performs well in most circumstances we tested, but it is outperformed by a naïve model during periods of low mosquito activity. This suggests the potential for combining results from multiple forecasting methods to achieve more reliable forecasts. Future efforts should focus on expanding the range of tested approaches, including compartmental models, machine learning, and statistical approaches (e.g., ARIMA). Nonetheless, the current tool represents a steppingstone toward improving situational awareness of mosquito control authorities, fulfilling the need for evidence-based decision making, and ultimately enhancing preparedness and response planning for mosquito-borne disease outbreaks.

## Methods

### Mosquito surveillance data

We have selected four jurisdictions in the US with highly different climatic conditions ranging from subtropical (Key West and Miami-Dade County, FL) to arid (Maricopa County, AZ) to temperate (Los Angeles County, CA). Moreover, these study sites have highly different populations in terms of income (median household income ranging from $67,000 in Miami-Dade County to $83,000 in Maricopa County), race/ethnicity (Miami-Dade County is predominantly Hispanic/Latino whereas the other three jurisdictions are predominantly White), and urbanization level (population ranging from about 25,000 residents in Key West to about 10 million residents in Los Angeles County) [34]. These locations also show stark differences in the temporal dynamics of *Ae*. *aegypti* population. Specifically, in Miami-Dade County and Key West, FL, *Ae*. *aegypti* is relatively abundant year-round whereas in Los Angeles County, CA, and Maricopa County, AZ, *Ae*. *aegypti* appears to be less abundant and with a much shorter season (Fig 2).

For Los Angeles County, CA, Maricopa County, AZ, Miami-Dade County, FL, mosquito surveillance data were provided by the local mosquito control authorities. For Key West, FL, the data is openly available and was retrieved from ref [35]. In all study sites, mosquitoes were collected by CO_2_-baited traps. In the study sites, trapping effort generally increased between years, but it was consistent within each year, except for Los Angeles County, which showed noticeable within-year variations. As such, for Los Angeles County, we analyzed only traps that were deployed for 20+ weeks per year. The resulting dataset includes an average of 51 deployed traps per week from 2018 to 2021 for Los Angeles County (range: 7–89), an average of 804 deployed traps per year between 2015 and 2021 for Maricopa County (range: 224–933), an average of 12 deployed traps per year from 2011 to 2020 (range: 8–13) for Key West, and an average of 255 deployed traps per year between 2019 and 2022 for Miami-Dade County (range: 165–413), see S1 Text. Overall, we analyzed data from 1357 traps. These traps were deployed for 24 hours once a week in shaded areas protected from direct solar radiation, wind, and precipitation to enhance mosquito collection. Mosquitoes were morphologically identified to species using taxonomic keys. The data is consistent between the four study sites, as they use the same type of trap and lure and have similar sampling efforts and frequency of data collection. Traps baited with CO_2_ are intended to lure female mosquitoes in search of a blood meal. As a result, male mosquitoes collected during this project were deemed incidental catches and excluded from this study.

Our forecast target is the relative abundance of female *Aedes aegypti*, which is defined as the number of collected specimens per trap night. All the data is spatially aggregated at the jurisdiction level (i.e., county, in the case of Los Angeles County, Maricopa County, and Miami-Dade County, or city, in the case of Key West). In other words, for each week in a given study site, we considered the total number of collected specimens in all traps divided by the number of traps deployed in that week.

### Temperature data

For each study site, we used temperature data from an online historical archive [36] that was collected by the international airport nearest to the study location. Average daily temperatures were used to estimate the temperature-dependent development and mortality rates for *Ae*. *aegypti* for each study location and day of the study period.

### Estimation of the time-dependent generation time

To obtain time-dependent estimates of *Ae*. *aegypti* generation time, we developed a compartmental chain-binomial model, which simulates *Ae*. *aegypti* population dynamics over time. In the model, the mosquito population is divided into four development stages: eggs (*E*), larvae (*L*), pupae (*P*), and female adults (*A*). Moreover, we consider a further compartment *A*_*E*_ that keeps track of the number of new eggs.

At the beginning of each simulation (time *t*^⋆^), we start with a fixed number of eggs (*E*(*t*^⋆^) = 1000). These transitions will be simulated through sampling from binomial distributions according to the development ($d_{E}(T),d_{L}(T),d_{P}(T),d_{A}(T)$) (note that for adults, *d*_*A*_ corresponds to the oviposition rate) or mortality ($m_{E}(T),m_{L}(T),m_{P}(T),m_{A}(T)$) rates at temperature *T* for each mosquito life stage, as taken from the literature [37].

The model is regulated by the following equations:

$$E(t+\Delta t)\leftarrow -m_{E}(\hat{T}(t))\cdot E(t)-d_{E}(\hat{T}(t))\cdot E(t)$$ (1)

$$L(t+\Delta t)\leftarrow d_{E}(\hat{T}(t))\cdot E(t)-m_{L}(\hat{T}(t))\cdot L(t)-d_{L}(\hat{T}(t))\cdot L(t)$$ (2)

$$P(t+\Delta t)\leftarrow d_{L}(\hat{T}(t))\cdot L(t)-m_{P}(\hat{T}(t))\cdot P(t)-d_{P}(\hat{T}(t))\cdot P(t)$$ (3)

$$A(t+\Delta t)\leftarrow {0.5\cdot d}_{P}(\hat{T}(t))\cdot P(t){-m}_{A}(\hat{T}(t))\cdot A(t)$$ (4)

$$A_{E}(t+\Delta t)\leftarrow A_{E}(t)+n_{E}d_{A}(\hat{T}(t))\cdot A(t)$$ (5)

where the arrow (←) represents the chain binomial process, *Δt* = 0.1 days is the time step used to simulate the chain binomial process, and $\hat{T}(t)$ is the temperature at calendar date *t* in the study location, and *n*_*E*_ represents the average number of eggs laid per oviposition.

As we are interested in estimating the generation time (instead of modeling the dynamics of the mosquito population), the new eggs laid by adult females are not introduced in the equation regulating the number of eggs (i.e., the equation for *E*(*t*)). Therefore, at a certain time step *t*^*f*^, the number of eggs will reach 0. Once the simulation is completed (i.e., *E*(*t*^*f*^) = 0), we estimated the distribution of the generation time for time *t*^⋆^, *T*_*g*_(*t*^⋆^), as

$$T_{g}(t^{⋆})=A_{E}(t^{⋆})/\sum_{t\in \{t^{⋆},\ldots ,t^{f}\}}A_{E}(t)$$ (6)

Finally, we fitted *T*_*g*_(*t*^⋆^) with a gamma distribution *ϕ*(*t*) to estimate its shape and rate parameters. *ϕ*(*t*) will be used in the Bayesian estimation of the population reproduction number over time (see next section). This procedure was run for all four study sites and dates.

### Estimation of the population reproduction number

First, we eliminated transient fluctuations from the mosquito surveillance data by applying a lowpass filter to the weekly number of collected *Ae*. *aegypti*. The cutoff frequency of the lowpass filter is given by *f*_*c*_ = *γ*_*c*_ *f*_*s*_, where *γ*_*c*_ is called the *relative cutoff* 0<*γ*_*c*_<1, and *f*_*s*_ = 0.5(day)^−1^ is the Nyquist frequency [38] of the data. Subsequently, we used a spline interpolation technique to generate the daily time series of mosquito relative abundance based on the filtered weekly data (see S1 Text). After that, we multiplied the data by a constant factor *S*>1 and rounded the resulting values to the nearest integers, translating the preprocessed rate (real numbers) into numbers of specimens (integer values). The relative cutoff *γ*_*c*_ and the data scaling factor *S* are both determined during the calibration process for each study site (see section Calibration of the forecasting tool). Finally, to prevent instabilities in the following steps, we replace zero and negative values (possible due to the interpolation and filtering) by 1.

Second, we used a Bayesian approach traditionally used to estimate the epidemic reproduction number [39–43] to estimate the reproduction number of *Ae*. *aegypti* population over time, *R*(*t*), based on the daily time series of *Ae*. *aegypti* relative abundance. Specifically, we explored the likelihood of observing the number of mosquitoes at calendar date *t* given a certain value of the reproduction number *R*(*t*), defined in the following equation:

$$\Lambda (t)=P(C(t),R(t)\sum_{s\in \{1,\ldots ,t\}}\phi (s)C(t-s)),$$ (7)

where *C*(*t*) indicates the (lowpass-filtered) relative abundance of mosquitoes on calendar date *t*, *ϕ*(*s*) is the distribution of the generation time at time *s* (see previous section), and *P*(*x*,*k*) represents the Poisson probability mass function evaluated at point *x* with parameter *k*. This likelihood was explored using Metropolis–Hastings Markov Chain Monte Carlo (MCMC) [39]. This MCMC procedure produces an ensemble of *N*_*MC*_ estimates of *R*(*t*) for each calendar date *t*.

### Estimation of the temporal trend of the reproduction number

For each study site, we divided the mosquito surveillance data into two datasets (Dataset 1 and Dataset 2) each one containing roughly the same number of seasons (see Table A in S1 Text). First, we relied on Dataset 1 to estimate *R*(*t*) for each study site and year of data. Then, by combining the estimates of the distribution of the mosquito population reproduction number for each day of the year *d*∈{1,…,365}, we obtained an estimate of the temporal trend for *R*_*h*_(*d*). In this case, forecasts relying on *R*_*h*_(*d*) estimated using Dataset 1 were validated against mosquito surveillance data from Dataset 1. Second, we repeated the same procedure by swapping Dataset 1 with Dataset 2.

### Forecast of *Ae*. *aegypti* relative abundance

To forecast *Ae*. *aegypti* relative abundance (i.e., the daily number of collected mosquitoes) in the 1- to 4-week period starting from the forecast date *τ*, we relied on the renewal equation [37,38]:

$$\overset{˘}{C}(t)=Pois(\overset{˘}{R}(t)[\sum_{s=1}^{\tau }\phi (s)C(t-s)+\sum_{s=\tau +1}^{t}\bar{\phi }(\tau )\overset{˘}{C}(t-s)]),$$ (8)

where $\overset{˘}{C}(t)$ represents the forecasted number of mosquitoes that will be collected at time *t*, $\overset{˘}{R}(t)$ represents the forecasted value of the population reproduction number at time *t*, $\bar{\phi }(\tau )$ is the average of the generation time in the 7 days before the forecast date (based on the mean and standard deviation of *ϕ*(*s*) in each day *s*), and all other parameters are as defined above.

To forecast the reproduction number $\overset{˘}{R}(t)$, we first calculated the average of the population reproduction number $\langle R(\tau )\rangle =\frac{1}{n}\sum_{s=1}^{n}R(\tau -s)$ and the historical reproduction number $\langle R_{h}(d_{\tau })\rangle =\frac{1}{n}\sum_{s=1}^{n}R_{h}(d_{\tau }-s)$ over the *n* = 7 days before the forecast. The average was separately calculated for each quantile of the ensembles. Second, we combined each sample of the historical and population reproduction number using the following expression:

$$\overset{˘}{R}(t)=\frac{\left\langle R(\tau )\right\rangle }{\left\langle R_{h}(d_{\tau })\right\rangle }R_{h}(d_{t}),$$ (9)

Where *d*_*t*_ is the day of the year corresponding to calendar date *t*. Then, each $\overset{˘}{R}(t)$ sample was used into the renewal equation to produce one forecasted trajectory of the number of mosquitoes $\overset{˘}{C}(t)$. Subsequently, we calculated the average of $\overset{˘}{C}(t)$ between each Sunday and the following Saturday, then divided by the scaling factor *S* applied during the preprocessing stage, obtaining the weekly number of mosquitoes collected per week and per trap night. Finally, to incorporate uncertainty due to random fluctuations, we applied a Gaussian noise to each weekly trajectory with mean 0 and standard deviation as estimated by comparing historical empirical data with the noise-filtered time series.

### Metrics for forecast evaluation

We employed a variety of metrics to assess the performance of the forecasts. The median forecast was used to calculate the symmetric Absolute Percentage Error (sAPE). For each observation *x*_*i*_ and a median forecast *m*_*i*_, sAPE_*i*_ is defined as:

$$\mathrm{sAP}E_{i}=2\cdot \frac{\left|m_{i}-x_{i}\right|}{\left(\left|m_{i}|+|x_{i}\right|\right)}.$$ (10)

This metric is symmetric between the forecast and observation, and it is bounded between 0 and 2, with lower values representing more accurate forecasts. Averaging this score over a set of observation-forecast pairs produces the symmetric Mean Absolute Percentage Error (sMAPE).

A second metric that we used to evaluate the forecasts is the coverage. Specifically, the *α forecast coverage* is given by the fraction of all observations that lie between the (*α*/2) and the (1−*α*/2) forecast quantiles. By interpreting the *α*–IQR as a Bayesian probability that the forecast encompasses the observation, the *α* coverage should ideally be equals to *α*. Therefore, we evaluate our forecast by calculating the *α coverage error*, given by the absolute difference between the *α* coverage and *α* itself, with zero representing an optimal coverage.

Finally, the third metric that we used is the Weighted Interval Score (WIS) [14]. The WIS is a weighted average of the performance of each IQR forecast, which assesses both the precision and the accuracy of a forecast, and is used as the main metric to evaluate forecast performances during CDC infectious disease forecast initiatives [11].

### Calibration of the forecasting tool

We calibrated our forecasting tool based on the WIS and the coverage error in a two-step procedure. For each study site, we selected three seasons for the calibration: 2018, 2019 and 2020 for Los Angeles County and for Maricopa County; 2016, 2017 and 2018 for Key West; 2019, 2020 and 2021 for Miami-Dade County. In the first calibration step, we performed a grid search over values of the relative filter cutoff *γ*_*c*_ in the [0,1] range with steps of size 0.005. The *γ*_*c*_ value which produced a forecast with the smallest WIS value was selected for the next step. In the second calibration step, we performed another grid search over values of the scaling factor *S* in the range [2,100], with steps of size 1.0. The value of *S* that provided the smallest value of $E=\sqrt{CE_{50\%}^{2}+CE_{95\%}^{2}}$ was selected, where $CE_{\alpha }^{2}$ is the *α* coverage error as described in the previous section. The values of *γ*_*c*_ and *S* selected in each of these steps were used to forecast the remaining seasons in each study site. The reason for using a two-step procedure instead of a single joint search is that the relative cutoff *γ*_*c*_ showed strong influence in the forecast accuracy but little influence in the coverage, while the scaling factor *S* had strong influence in the coverage but weak influence in the accuracy (see Fig A in S1 Text). The parameter values obtained through this procedure are reported in Table B in S1 Text for each study site.

### Naïve forecasting model

To provide a reference performance for our forecasting tool, we implemented a “naïve” model based on past differences in the target data was implemented. Specifically, we adapted an approach commonly used in CDC infectious disease forecast initiatives [11]. Given the ground truth data in a study site, we first calculated the positive and negative difference series up to the forecast date, corresponding to the difference between the mosquito relative abundance in that week subtracted by the relative abundance in the previous week. The same value with inverted signal is also accounted for the final ensemble. From this ensemble of differences, we fit a normal distribution with mean zero. This distribution is then used to create forecast trajectories, each one given by *c*(*t*+1) = *c*(*t*)+*ϵ*(*t*), where *ϵ*(*t*) is a value sampled from the fitted normal distribution, and *c*(*t*) is the relative *Ae*. *aegypti* abundance for week *t*. For each study site and forecast date, a total of 1,000 trajectories were generated using this method, from which the quantile-based forecasts were constructed.

## Supporting information

S1 Text Details on the methodology and additional results. (PDF)
