# Supplementary material for: Forecasting the relative abundance of Aedes vector populations to enhance situational awareness for mosquito control operations
Source: PLoS Negl Trop Dis. 2024 Nov 25;18(11):e0012671. doi: 10.1371/journal.pntd.0012671 (PMC11627370; doi:10.1371/journal.pntd.0012671)
Supplement: S1 Text — (PDF) [file pntd.0012671.s001.pdf]

Forecasting the relative abundance of Aedes vector populations to enhance situational awareness for mosquito control operations

Supplementary Information

Paulo C. Ventura[1], Allisandra G. Kummer[1], André B. B. Wilke[1], Jagadeesh Chitturi[1], Megan D. Hill[1], Chalmers Vasquez[2], Isik Unlu[2], John-Paul Mutebi[2], Susanne Kluh[3], Steve Vetrone[3], Dan Damian[4], John Townsend[4], Maria Litvinova[1], Marco Ajelli[1,$]

[1]Laboratory for Computational Epidemiology and Public Health, Department of Epidemiology and Biostatistics, Indiana University School of Public Health, Bloomington, Indiana, USA

[2]Miami-Dade County Mosquito Control Division, Miami, Florida, USA

[3]Greater Los Angeles County Vector Control District, Santa Fe Springs, California, USA

[4]Maricopa County Environmental Services Department, Vector Control Division, Phoenix, AZ, USA

[$]Corresponding author: majelli@iu.edu

S1. Methodology details

S1.1. Spline interpolation to infer daily abundance from weekly data

For all considered study sites, we have weekly reported Aedes aegypti relative abundance data, represented by the number of collected specimens per trap night. From our compartmental model estimates, we know that the generation time of *Ae aegypti* is typically between 5 and 20 days, but can be as low as 3 days. This means that using weekly data to estimate the reproduction number R(t) could lead to suboptimal results, due to lack of temporal resolution. Therefore, we used an interpolation technique to infer daily-resolution abundance from weekly time series.

The method assumes that each weekly observation represents the Saturday of that week, and that the observations change smoothly between consecutive observations (Saturdays). Importantly, we perform this interpolation *after the lowpass filter* is applied to the raw data, removing short-term oscillations from the raw time series. We used a cubic spline interpolation to fill the values between weekly observations at a daily resolution.

S1.2. Training and testing datasets

In order to calculate the historical temporal trends of the reproduction number, we divided the mosquito surveillance data of each study site into two datasets (Dataset 1 and Dataset 2) each one containing roughly the same number of seasons. The seasons that were put into each dataset are shown in Table A:

Table A. Splitting of the mosquito surveillance between two sets of seasons for each study site.

Study site	Dataset 1	Dataset 2
**Los Angeles County**	2018, 2019	2020, 2021
**Maricopa County**	2015, 2016, 2017	2018, 2019, 2020, 2021
**Key West**	2011, 2012, 2013, 2014, 2015	2016, 2017, 2018, 2019, 2020
**Miami-Dade County**	2019, 2020	2021, 2022

S2. Additional results

S2.1. Model calibration

For the model calibration, we performed a two-step grid search over two parameters that affect the forecast performance: the relative lowpass filter cutoff (γ_c) and the scaling factor applied to the data before the forecast (S). As described in the main text, the first step selected the relative cutoff that minimized the WIS, and the second step selected the scaling factor that minimized the 50% and 95% quadratic coverage errors. Changing the scaling factor after choosing a cutoff value had little effect over the WIS, allowing the calibrated forecasts to have both good accuracy and coverage even for the seasons not used in the calibration (Fig A). The optimal parameters, as determined by the two-step calibration for each location, are shown in Table B.

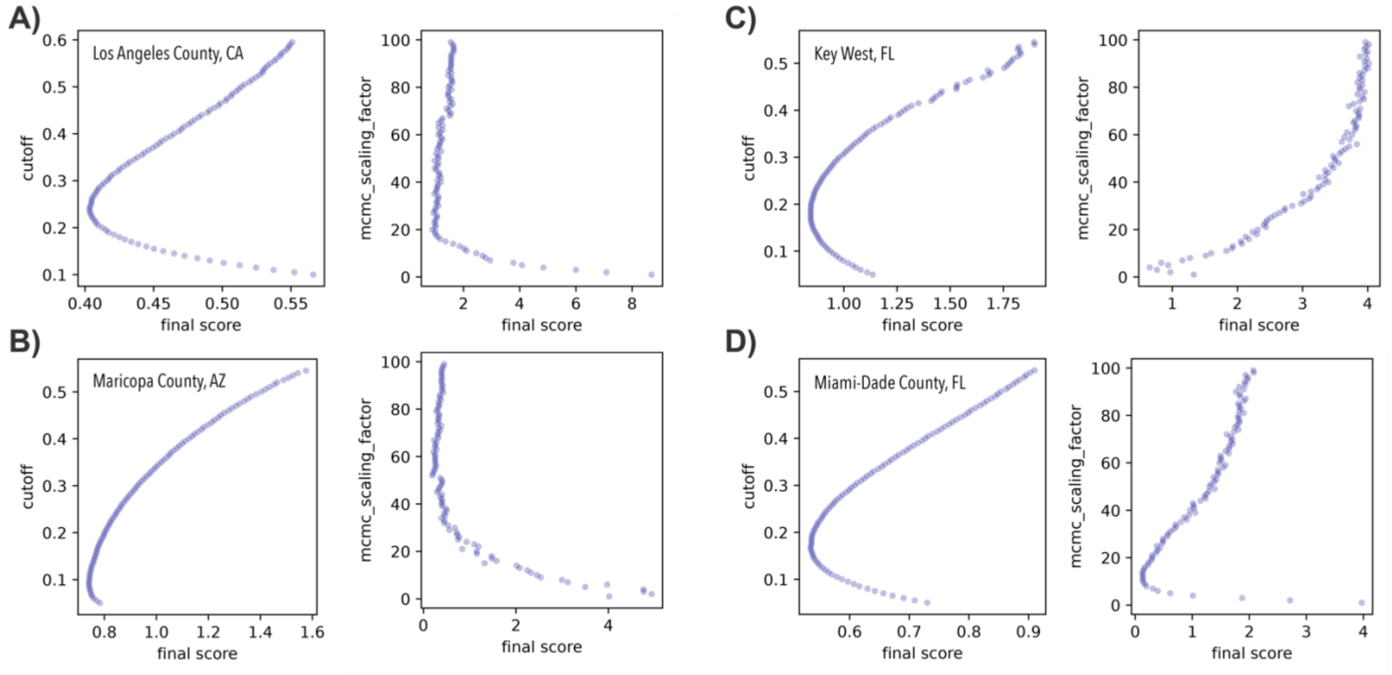

Fig A. Results of the parameter search for each location. A) Los Angeles County, CA, B) Maricopa County, AZ, C) Key West, FL, D) Miami-Dade County, FL. The left panels represent the first step of the calibration, with the WIS normalized by the average incidence over the season shown in the x axis and the relative cutoff γ_c shown in the y axis. The right panels represent the results of the second step, with the sum of the quadratic 50% and 95% coverage errors in the x axis (arbitrary units) and the scaling factor S in the y axis.

Table B. Optimal parameters obtained from the model calibration for each study site.

**Study site**	**Relative filter cutoff (γ_c)**	**Scaling factor (S)**
**Los Angeles County**	0.240	20
**Maricopa County**	0.095	52
**Key West**	0.185	4
**Miami-Dade County**	0.165	13

S2.2. Forecast horizon time series

In this work, we produced forecasts for weekly horizons between 1 and 4 weeks ahead of each reference date. In the main manuscript, we showed the 2 weeks horizon median and 90% IQR forecasts over time for the (Fig 2

in the main text). Here we show the same plots for the 1, 3 and 4 weeks horizons (Figs B, C and D respectively). By comparing the different horizons, we can see how the forecast evolves over the horizon time.

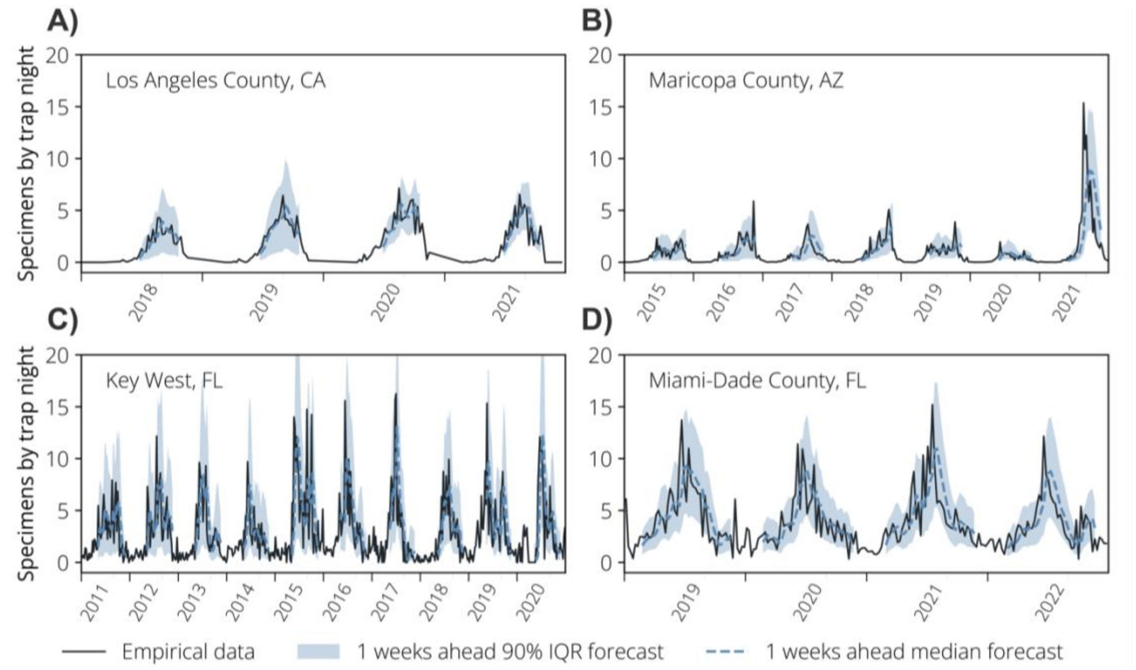

Fig B. **Mosquito relative abundance over time – 1 week horizon. A** Observed and forecasted number of collected specimens per trap night over time in Los Angeles County, CA. The solid line represents the number of *Ae. aegypti* collected by the mosquito surveillance system per week, the dashed line represents the median of the first week of the 1-week ahead forecast, and the shaded areas are the 90% IQR of the first week of the 1-week ahead forecast. **B** As A, but for Maricopa County, AZ. **C** As A, but for Key West, FL. **D** As A, but for Miami-Dade County, FL, respectively.

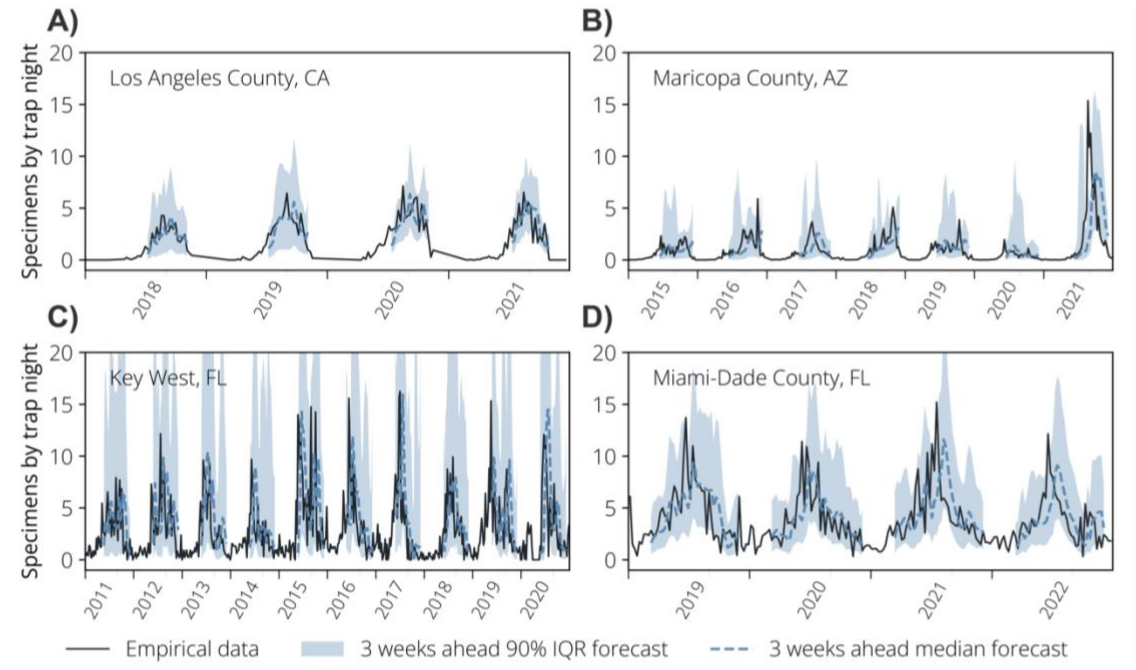

Fig C. **Mosquito relative abundance over time – 3 weeks horizon. A** Observed and forecasted number of collected specimens per trap night over time in Los Angeles County, CA. The solid line represents the number of *Ae. aegypti* collected by the mosquito surveillance system per week, the dashed line represents the median of the third week of the 3-week ahead forecast, and the shaded areas are the 90% IQR of the third week of the 3-week ahead forecast. **B** As A, but for Maricopa County, AZ. **C** As A, but for Key West, FL. **D** As A, but for Miami-Dade County, FL, respectively.

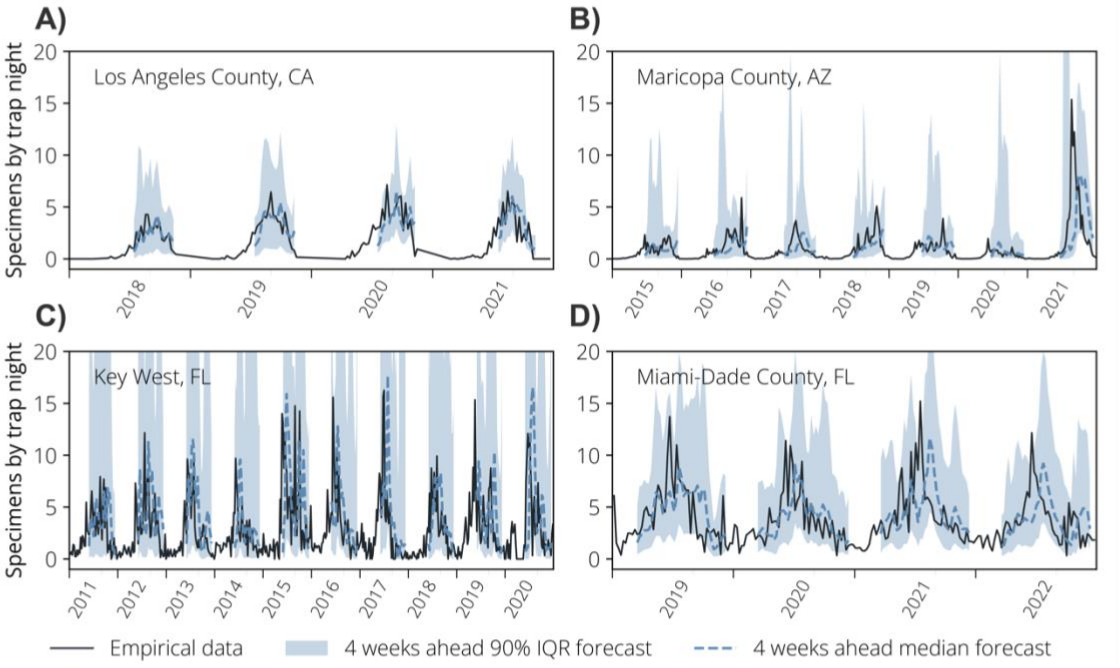

Fig D. **Mosquito relative abundance over time – 4 weeks horizon. A** Observed and forecasted number of collected specimens per trap night over time in Los Angeles County, CA. The solid line represents the number of *Ae. aegypti* collected by the mosquito surveillance system per week, the dashed line represents the median of the fourth week of the 4-week ahead forecast, and the shaded areas are the 90% IQR of the fourth week of the 4-week ahead forecast. **B** As A, but for Maricopa County, AZ. **C** As A, but for Key West, FL. **D** As A, but for Miami-Dade County, FL, respectively.

S2.3. Forecast scores

Scoring metrics calculated independently for each season show how the performance of our analytical tool varies with the specificities of each location and season. The Weighted Interval Score (WIS)[1], which account for both the precision and accuracy of the forecasts, was used to calibrate the model for each location; its season-normalized value (i.e., divided by the average incidence during the forecast season) ranged from 0.96 to 2.89, obtained respectively for the 2019 Los Angeles season and the 2021 Maricopa season. The WIS is split into two components: calibration and sharpness. The sharpness measures the width of the prediction intervals, regardless of whether they contain the observation or not, and can be used to assess the precision of forecasts. The 95% IQR forecast achieved 100% coverage in four settings: Los Angeles 2018 and 2019, Key West 2013 and 2014. The minimum coverage of the 95% IQR forecast was obtained for Los Angeles in 2021 at 79.4%. The 95% IQR forecast reached a maximum of 65.3% for Los Angeles in 2018 and a minimum of 27.9% for Maricopa 2021. The symmetric mean absolute percentage error (sMAPE) score ranged from 0.293 (Los Angeles 2019) to 0.90 (Key West 2017), with smaller values representing more accurate median forecasts (Table C). The performance of our analytical tool can also be compared with that of the naïve forecast model (Table D).

Table C. Evaluation scores for our analytical tool, for all seasons in each study site.

Study site	Season	Season normalized WIS	95% coverage	sMAPE score	Season normalized sharpness
Los Angeles	2018	1.051	100.0%	0.316	0.648
	2019	0.956	100.0%	0.293	0.587
	2020	1.052	88.2%	0.329	0.357
	2021	1.207	79.4%	0.424	0.398
Maricopa	2015	1.955	95.2%	0.576	0.883
	2016	1.780	93.3%	0.479	0.699
	2017	2.234	97.0%	0.599	0.886
	2018	1.759	91.0%	0.528	0.570
	2019	2.082	90.0%	0.532	0.861
	2020	2.260	96.2%	0.519	1.337
	2021	2.886	80.8%	0.802	0.568
Key West	2011	2.116	95.7%	0.587	1.239
	2012	2.315	95.5%	0.617	1.189
	2013	2.355	100.0%	0.672	1.341
	2014	2.530	100.0%	0.630	1.339
	2015	2.393	94.8%	0.645	0.985
	2016	2.211	94.8%	0.564	1.072
	2017	2.706	81.3%	0.901	1.240
	2018	2.030	96.4%	0.573	1.212
	2019	2.384	99.1%	0.622	1.147
	2020	2.790	95.7%	0.695	1.326
Miami-Dade	2019	1.268	91.0%	0.397	0.523
	2020	1.298	93.8%	0.371	0.554
	2021	1.475	94.2%	0.384	0.539
	2022	1.480	89.7%	0.462	0.556

Table D. Evaluation scores for our analytical tool, for all seasons in each study site.

Study site	Season	Season normalized WIS	95% coverage	sMAPE score	Season normalized sharpness
Los Angeles	2018	1.603	65.3%	0.460	0.102
	2019	1.368	58.3%	0.387	0.076
	2020	1.451	60.3%	0.362	0.091
	2021	1.808	52.9%	0.530	0.182
Maricopa	2015	2.116	82.7%	0.544	0.108
	2016	2.460	72.1%	0.485	0.045
	2017	1.863	82.0%	0.455	0.235
	2018	2.326	62.0%	0.554	0.130
	2019	2.175	83.0%	0.503	0.185
	2020	1.948	94.2%	0.433	0.302
	2021	2.675	49.0%	0.579	0.110
Key West	2011	2.119	87.9%	0.675	0.656
	2012	2.248	84.8%	0.646	0.556
	2013	2.114	85.7%	0.595	0.617
	2014	2.401	89.3%	0.688	0.718
	2015	2.697	66.4%	0.690	0.406
	2016	2.394	86.2%	0.641	0.694
	2017	2.759	84.8%	0.882	0.694
	2018	2.078	88.4%	0.682	0.740
	2019	2.545	85.7%	0.646	0.727
	2020	2.482	81.9%	0.790	0.613
Miami-Dade	2019	1.473	87.2%	0.402	0.433
	2020	1.680	89.4%	0.462	0.587
	2021	1.618	86.5%	0.385	0.512
	2022	1.501	93.6%	0.492	0.587

S2.4. *Aedes aegypti* collection and trapping effort data

As explained in the main text, the relative abundance of *Ae. aegypti* in our study sites was measured by collecting specimens with baited traps spread throughout the jurisdiction areas. These traps were deployed according to different criteria in each study site, depending on the goals and interests of the local authorities. We therefore observe diverse patterns of trapping efforts in each dataset, which also has implications to the mosquito abundance data. We reduced the effects of these patterns by dividing the total number of collected specimens by the number of traps deployed in each week, but some residual effects may still be in place. The most expressive example is Los Angeles County, CA, for which the trapping effort significantly changes during the *Ae. aegypti* season. We report the target data, number of species divided by the number of deployed traps, and the underlying trapping efforts (number of deployed traps) in Fig S5.

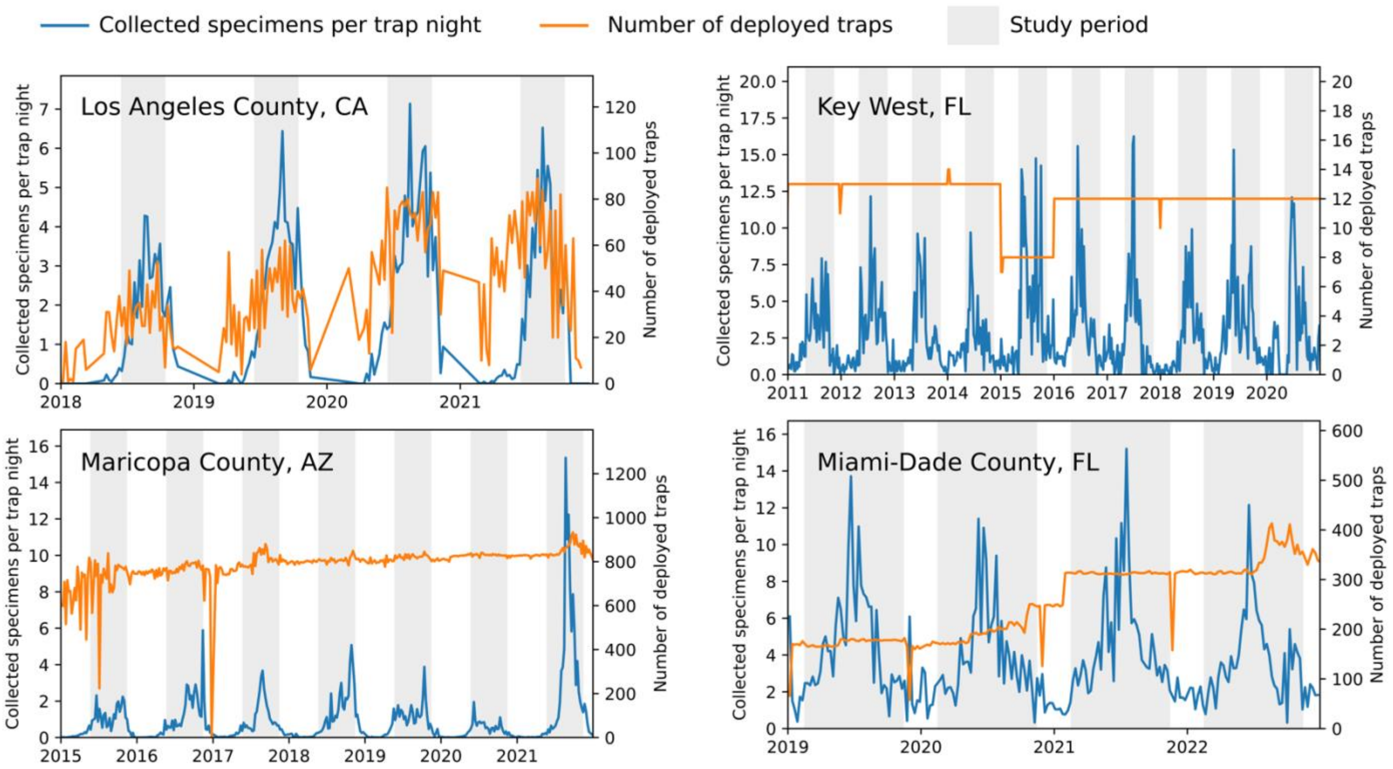

Fig S5. Target data and trapping efforts for the considered study sites. The blue line, scaled by the left-hand y-axis, represents our forecast target data, which is the total number of collected *Ae. aegypti* specimens during each week divided by the number of traps that were deployed for 24h during that same week. The orange line, scaled by the right-hand y-axis, represents the number of traps deployed over 24h periods during each week, which is the denominator term of the target data. For Los Angeles County, only traps that were deployed 20+ times per year are considered. The gray shaded areas represent the study periods that we considered for each jurisdiction.

References

1. Bracher, J., Ray, E. L., Gneiting, T. & Reich, N. G. Evaluating epidemic forecasts in an interval format. *PLOS Computational Biology* **17**, e1008618 (2021).
